# Relationship between gastrointestinal symptoms and COVID-19 infection in the pediatric population: a scoping review

**DOI:** 10.1590/1980-220X-REEUSP-2023-0365en

**Published:** 2024-05-06

**Authors:** Denise Desconsi, Juliane Pagliari Araujo, Marcela Demitto Furtado, Rosângela Aparecida Pimenta, Adriana Valongo Zani

**Affiliations:** 1 Universidade Estadual de Londrina, Londrina, PR, Brazil.; 2 Universidade Estadual de Maringá, Maringá, PR, Brazil.

**Keywords:** Child, Infant, Newborn, Gastrointestinal Tract, COVID-19, Niño, Recién Nacido, Tracto Gastrointestinal, COVID-19, Criança, Recém-Nascido, Trato Gastrointestinal, COVID-19

## Abstract

**Objective:**

To map the evidence in the literature about the relationship between gastrointestinal symptoms and COVID-19 in the pediatric population.

**Method:**

This is a scoping review following the recommendations of the Joanna Briggs Institute and PRISMA Extension for Scoping Reviews (PRISMA-ScR): Checklist and Explanation. The search was carried out on the following bases: Embase, Google Scholar, PubMed, Scopus, LILACS, CINAHL, Scielo, Web of Science and Virtual Health Library Portal, between July and August 2023. Original studies available in full, in any language, were included.

**Results:**

Ten studies were chosen that pointed to three premises: (1) the ACE2 receptor is found in the epithelial cells of the gastrointestinal tract; (2) gastrointestinal symptoms are mediated by stress and infection is justified by the gut-brain axis; (3) it develops the process of Multisystem Inflammatory Syndrome in children, affecting the gastrointestinal tract.

**Conclusion:**

The synthesis of evidence provided three assumptions which guide the origin of gastrointestinal symptoms. The identification of gastrointestinal symptoms in children affected by COVID-19 can assist in the clinical approach and management of care and treatments.

## RESUMO

**Objetivo:**

Mapear as evidências na literatura acerca da relação entre sintomas gastrointestinais e a COVID-19 na população pediátrica.

**Método:**

Trata-se de *scoping review* seguindo as recomendações do Instituto Joanna Briggs e PRISMA Extension for Scoping Reviews (PRISMA-ScR): Checklist and Explanation. A busca foi realizada nas bases: Embase, Google Acadêmico, PubMed, Scopus, LILACS, CINAHL, Scielo, Web of Science e Portal da Biblioteca Virtual em Saúde, entre julho e agosto de 2023. Foram incluídos estudos originais disponíveis na íntegra, em qualquer idioma.

**Resultados:**

Foram eleitos 10 estudos que apontaram para três premissas: (1) o receptor ECA 2 é encontrado nas células epiteliais do trato gastrointestinal; (2) os sintomas gastrointestinais são mediados pelo estresse e a infecção é justificada pelo eixo cérebro-intestino; (3) desenvolve o processo de Síndrome Inflamatória Multissistêmica em crianças, afetando o trato gastrointestinal.

**Conclusão:**

A síntese de evidências proporcionou três pressupostos os quais orientam a origem dos sintomas gastrointestinais. A identificação dos sintomas gastrointestinais em crianças acometidas por COVID-19 pode auxiliar na abordagem clínica e manejo de cuidados e tratamentos.

## RESUMEN

**Objetivo:**

Mapear la evidencia en la literatura sobre la relación entre síntomas gastrointestinales y COVID-19 en la población pediátrica.

**Método:**

Se trata de revisión de alcance siguiendo las recomendaciones del Instituto Joanna Briggs y PRISMA Extension for Scoping Reviews (PRISMA-ScR): Lista de verificación y explicación. La búsqueda se realizó sobre las siguientes bases: Embase, Google Scholar, PubMed, Scopus, LILACS, CINAHL, Scielo, Web of Science y Portal Biblioteca Virtual en Salud, entre julio y agosto de 2023. Se incluyeron estudios originales disponibles en su totalidad, en cualquier idioma.

**Resultados:**

Se eligieron 10 estudios que apuntaban a tres premisas: (1) el receptor ACE2 se encuentra en las células epiteliales del tracto gastrointestinal; (2) los síntomas gastrointestinales están mediados por el estrés y la infección está justificada por el eje cerebro-intestino; (3) desarrolla el proceso del Síndrome Inflamatorio Multisistémico en niños, afectando el tracto gastrointestinal.

**Conclusión:**

La síntesis de evidencia proporcionó tres supuestos que guían el origen de los síntomas gastrointestinales. La identificación de síntomas gastrointestinales en niños afectados por COVID-19 puede ayudar en el abordaje clínico y el manejo de la atención y los tratamientos.

## INTRODUCTION

Global public health faces challenges with new infectious diseases and other existing diseases^(1)^. Respiratory diseases comprise a broad spectrum, affecting the structures of the upper and lower respiratory systems^(2)^, thus leading to the main causes of hospitalizations, morbidities, and mortalities in the world^(3)^. In the childhood scenario, respiratory system disorders represent one of the main causes of morbidity and mortality in children under 5 years of age^(4)^.

Brazil presented, between 2012 and 2021, around 22,939 deaths, related to the respiratory system, in children under 10 years of age, and 62.65% of these were in the first year of life^(5)^. Furthermore, given the consequences of emerging diseases, in 2019 a new disease belonging to the *Coronaviridae* family was discovered, called COVID-19, which mainly affects the respiratory system^(6)^. Given this new discovery, restrictive measures, such as social distancing, were imposed to control the spread of the virus, as there was a lack of scientific knowledge for treatment and prevention^(7)^.

At the moment, there are six registered varieties of Coronavirus, namely 229E, OC43, NL63, HKU1, and these four cause flu-like symptoms in people; SARS-CoV (Severe Acute Respiratory Syndrome or SARS) and MERS-CoV (Middle East Respiratory Syndrome or MERS) have the ability to cause severe respiratory syndrome with high fatality rates^(8)^. SARS-CoV-2 (Coronavirus Severe Acute Respiratory Syndrome 2) is called Novel Coronavirus-Infected Pneumonia (NCIP) and is part of the betacoronavirus group that encompasses SARS-CoV. It was analyzed and confirmed in bronchoalveolar lavage and, later, genetic sequencing, Polymerase Chain Reaction (PCR) and culture were performed on the first infected patients in Wuhan, China, in 2019^(9)^.

Therefore, at the beginning of the contamination, whole genome sequence analyses, culture of human airway epithelial cells and microscopy were carried out, considering respiratory secretions^(1)^ as the predominant means of transmission of the virus. Thus, the child population is exposed to transmission of the virus, which can take place both through direct contact with infected people and through indirect contact with infected surfaces^(10)^.

The epidemiological and clinical characteristics of children infected with SARS-CoV-2 may be asymptomatic, presenting mild symptoms such as fever, cough, runny nose, headache, nausea, vomiting, and diarrhea^(11,12,13,14)^, or serious impairment, such as respiratory failure, pneumonia, and multiple organ failure^(15,16)^. Among the symptoms presented by children, fever is the most common one; however, gastrointestinal symptoms have a high incidence in this population. Around 90% of care provided to the pediatric population with exposure to or diagnosis of COVID-19 presented gastric or intestinal manifestations^(17,18,19)^. However, in some cases, only gastric and intestinal manifestations are observed, and there is an absence of respiratory manifestations^(20)^. Tests carried out showed negative nasopharyngeal results and positive rectal swab, thus indicating that the gastrointestinal tract can release the virus and consequently fecal-oral transmission may be possible^(21)^.

In this regard, the pediatric population may only develop gastrointestinal symptoms, without presenting other clinical manifestations^(22)^. Furthermore, gastric and intestinal involvement is associated with greater severity of the disease^(20)^. It was found that SARS-CoV-2 is capable of replicating intestinal epithelial cells, thus developing tissue injuries and an increase in a large number of innate immune cells, triggering a deregulated hyperinflammatory response^(23,24)^.

Therefore, there is a need to review the clinical approach used to treat gastrointestinal symptoms in the pediatric population, as the digestive system is often not associated with SARS-CoV-2 viral infection^(25)^. Healthcare professionals should pay attention to acute gastrointestinal manifestations in the child population, considering COVID-19 as a differential diagnosis^(26,27)^, as there are extrapulmonary symptoms of COVID-19, involving the gastrointestinal system, recently detected. They are a cause for concern among health professionals who care for this population^(28,29)^.

Given this context, this study is warranted by the need to know and compile data on gastrointestinal symptoms in children, to provide data for clinical practice, thus reinforcing rapid diagnosis for the pediatric population. In addition, the digestive system can be a potential route of infection transmission by the new Coronavirus, and research on this topic can provide scientific evidence to support care for children with COVID-19. Thus, this study aimed at mapping the evidence in the literature about the relationship between gastrointestinal symptoms and COVID-19 in the pediatric population.

## METHOD

### Design of Study

This is a scoping review, which aims to map the current evidence available in the literature, as well as to present the main concepts in the area and show the gaps in knowledge available, allowing the exploration of new research^(30)^. Scoping reviews are complex studies, as they involve methodological rigor, and must be developed with independent evaluation involving at least two reviewers and following protocols^(31)^. This study was built based on the recommendations of the *Joanna Briggs* Institute (JBI) Review Manual and the PRISMA extension – *Extension for Scoping Reviews (PRISMA-ScR): Checklist and Explanation* ^(32,33)^. The research protocol has been published^(34)^ and registered with *Open Science Framework* with identifier https://doi.org/10.17605/OSF.IO/G59AB.

### Data Sources and Research Strategy

To construct this review, nine databases were accessed through the Periodical Portal of the Coordination for the Improvement of Higher Education Personnel (CAPES), including: *Excerpta Medica data BASE* (Embase), Google Scholar, PubMed, Scopus, Latin American and Caribbean Literature in Health Sciences (LILACS), *Cumulative Index to Nursing and Allied Health Literature* (CINAHL), Scielo, *Web of Science,* and the Virtual Health Library (VHL) website.

The review question was formulated according to the acronym PCC (Population, Concept and Context), with Population (P) being child, newborn, infant; Concept (C), gastrointestinal tract; Context (C), COVID-19. The research question was therefore: What is the relationship between gastrointestinal symptoms and COVID-19 in the pediatric population?

Moreover, MeSH (*Medical Subject Headings*), DeCS (Health Sciences Descriptors), and CINAHL *Headings* descriptors were used, together with boolean operators *AND* and *OR*. The search strategy presentation in each database is described in Chart 1.

**Chart 1:** Database search strategy – Londrina, PR, Brazil, 2023.

Base	Search Strategy	Results found
**EMBASE**	#1 child OR newborn AND “gastrointestinal tract” AND COVID-19 #2 child OR newborn OR infant AND “gastrointestinal tract” AND COVID-19 #3 infant premature OR child OR infant OR newborn AND “gastrointestinal tract” AND “SARS-CoV-2 infection”	110 78 79
**Google Scholar**	#1 infant premature OR child OR infant OR newborn AND “gastrointestinal tract” AND “SARS-CoV-2 infection” #2 lactente AND trato gastrointestinal AND infecção por SARS-CoV-2	2128 236
**PubMed**	#1 child OR newborn AND “gastrointestinal tract” AND COVID-19 #2 infant premature OR child OR infant OR newborn AND “gastrointestinal tract” AND “SARS-CoV-2 infection”	1866 1089
**Scopus**	#1 child OR newborn AND “gastrointestinal tract” AND COVID-19 #2 child OR newborn OR infant AND “gastrointestinal tract” AND COVID-19 #3 infant premature OR child OR infant OR newborn AND “gastrointestinal tract” AND “SARS-CoV-2 infection”	340 74 05
**LILACS**	#1 criança AND recém-nascido AND COVID-19 #2 criança OR recém-nascido AND COVID-19 #3 neonato AND trato gastrointestinal AND COVID-19 #4 criança recém-nascida AND trato gastrointestinal AND COVID-19 #5 recém-nascido AND aparelho gastrointestinal AND doença por Coronavírus 2019 #6 recém-nascido OR prematuro AND trato gastrointestinal AND COVID-19 #7 neonato OR recém-nascido AND trato gastrointestinal AND COVID-19 #8 recém-nascido OR criança AND tubo digestório AND infecção por SARS-CoV-2 #9 lactente AND trato gastrointestinal AND infecção por SARS-CoV-2 #10 lactente OR prematuro AND trato gastrointestinal AND COVID-19	35 0 0 0 1 0 3 0 4 0
**CINAHL**	#1 child AND “gastrointestinal tract” AND COVID-19 #2 child OR newborn OR infant AND “gastrointestinal tract” AND COVID-19 #3 newborn AND “gastrointestinal tract” AND COVID-19 #4 newborn AND “gastrointestinal tract” OR “digestive tube” AND COVID-19	16 120 1 80
**Scielo**	#1 infant premature OR child OR infant OR newborn AND “gastrointestinal tract” AND “SARS-CoV-2 infection” #2 child OR newborn OR infant AND “gastrointestinal tract” AND COVID-19 #3 child OR newborn AND “gastrointestinal tract” AND COVID-19	0 0 0
**Web of Science**	#1 child AND “gastrointestinal tract” AND COVID-19	77
**VHS**	#1 child AND “gastrointestinal tract” AND COVID-19 #2 infant premature OR child OR infant OR newborn AND “gastrointestinal tract” AND “SARS-CoV-2 infection” #3 child OR newborn OR infant AND “gastrointestinal tract” AND COVID-19	89 16 37

### Data Collection and Extraction

Data collection took place in July and August 2023. The eligibility criteria were primary studies and technical notes addressing the topic of gastrointestinal symptoms and COVID-19 and that answered the research question. Furthermore, no language or publication date limitations were established, considering the lack of studies related to the topic. Also, studies addressing newborns (NB), infants, children and adolescents, or adults in the same study were included, using data only from the pediatric population. Duplicate studies were counted once.

The studies were analyzed by two independent reviewers, who carried out an in-depth reading considering the eligibility criteria. A third reviewer was called in to resolve differences and thus avoid the risk of bias. All information, as well as captured studies, were stored in electronic spreadsheets and in analytical tables where the information was exposed for better interpretation and comparison of productions, thus allowing the description of the evidence.

After reading the titles and abstracts, the pre-selected studies underwent data analysis and mapping. For this, the Review Manual of the JBI^(23)^ was used, which consisted of careful reading and classification of texts extracting the results. A manual search took place by checking the list of references of the studies included in the review. This search yielded articles that were included in the final amount. This search aimed to find studies that were not identified in the databases.

The following data were extracted from the studies: authors/year, objective, population, main gastrointestinal symptoms, tests performed, association of gastrointestinal symptoms with COVID-19.

### Summary of Results

In the selected studies, a descriptive analysis of the variables was carried out, with critical analysis and discussion. The main characteristics of the selected studies were organized and presented in summary charts, containing the most important information, responding to the objective of the study, such as main gastrointestinal symptoms, tests performed and association of gastrointestinal symptoms.

### Ethical Aspects

This scoping review was carried out considering the ethical aspects regarding the authorship of the articles researched and selected, with all authors being duly cited. As it is a study performed exclusively with scientific texts, approval by a Research Ethics Committee is not required.

## RESULTS

**Figure 1:**
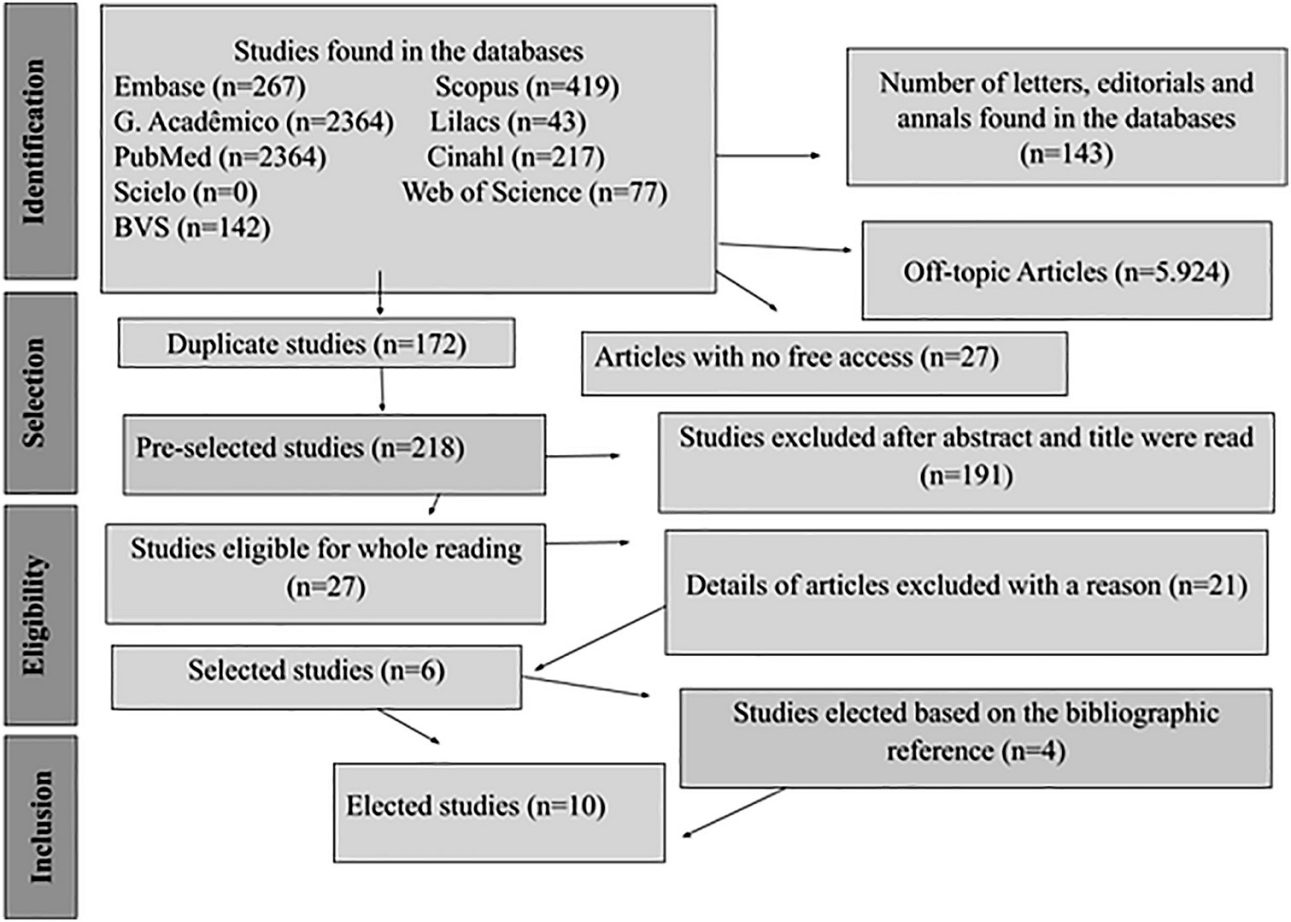
PRISMA-ScR flowchart of study selection and inclusion process in the scoping review – Londrina, PR, Brazil, 2023.

The search strategies allowed us to find a total of 6,484 publications, of which 172 were duplicates, 27 were not freely accessible, and 5,924 were outside the topic of this review. A total of 218 studies was selected for title and abstract reading and, after this process, 27 were selected for full reading, with 191 being excluded for not answering the objective and research question. Therefore, six studies were chosen to compose the present review. Additionally, the bibliographic references of these studies were explored and, after considering the eligibility criteria again, four more publications were added to compose the results, totaling 10 articles (Figure 1).

The studies included were published in 2020 (n = 05), 2021 (n = 03), 2022 (n = 01) and 2023 (n = 01), in different countries such as Italy (n = 03), United States of America (n = 1), Brazil (n = 01), Iran (n = 01), Romania (n = 01), China (n = 01), and Russia (n = 2). As for the language of publication, English was predominant.

In Chart 2 the authors, year of publication, main objective of the study, population, and type of study adopted in the articles that made up this research are demonstrated.

**Chart 2:** Characterization of selected studies regarding identification, author, year, objective, population, and type of study – Londrina, PR, Brazil, 2023.

Authors	Objective	Population	Design of study
Xu et al.^(35)^	To report the initial epidemiological and clinical features of SARS-CoV-2 infection tested for evidence of viral excretion through the gastrointestinal and respiratory tracts.	Children aged 0 to 18.	Case report
Miller et al.^(36)^	To evaluate the prevalence and presentations of gastrointestinal symptoms derived from pediatric multisystem inflammatory syndrome related to COVID-19.	Children between 7 months and 20 years old.	Retrospective
Giacomet et al.^(37)^	To describe the results of a preliminary analysis of a cohort of pediatric patients hospitalized with COVID-19 with a focus on mode of presentation, presence of comorbidities, disease severity, and early outcome.	Children between 1 and 18 years old.	Multicenter retrospective
Dooki et al.^(38)^	To determine the presentations of gastrointestinal and liver injuries in children admitted with COVID-19 infection to Amirkola Children’s Hospital.	Children between 2 months and 18 years old.	Retrospective
Souza et al.^(39)^	To highlight gastrointestinal manifestations as the initial presentation of SARS-CoV-2, through the analysis of pediatric patients followed in the pediatric intensive care unit of an emergency hospital, diagnosed with COVID-19.	Children aged 0 to 14.	Retrospective
Lo Vecchio et al.^(40)^	To describe the clinical, radiological and histopathological characteristics of children with COVID-19 presenting severe gastrointestinal manifestations.	Children under 18.	Multicenter retrospective cohort
Berni Canani et al.^(41)^	To evaluate the expression of the most relevant mediators of SARS-CoV-2 infection: ACE2, ACE1, TMPRSS2 and NRP1, in the upper respiratory tract and small intestine of children.	Children aged 1 to 10.	Prospective observational
Farello et al.^(42)^	To assess the impact of the COVID-19 pandemic on the prevalence of functional gastrointestinal disorders in Italian children and adolescents.	Children between 10 and 17 years old.	Cross-sectional with case report
Stepan et al.^(43)^	To investigate the functional disorders of abdominal pain in preschool children affected by a viral infection, according to the most recent diagnostic criteria.	Children aged 4 to 6 years.	Retrospective, observational descriptive and analytical
Vasichkin et al.^(44)^	To report clinical cases that demonstrate difficulties in diagnosing polyorganic disorders in children infected by SARS-CoV-2 at different periods of the disease.	Children aged 7 and 12.	Case report

* SARS-CoV-2: Severe acute respiratory syndrome coronavirus 2; ACE2: angiotensin-converting enzyme 2; ACE1: angiotensin-converting enzyme 1; TMPRSS2: transmembrane serine protease 2; NRP1: Neuropilin 1.

Chart 3 presents the main gastrointestinal symptoms, the tests carried out during the contagion period and the association of symptoms with COVID-19 presented by the populations of the selected studies.

**Chart 3:** Distribution of selected articles according to the description of gastrointestinal symptoms, tests performed, and their relationship with COVID-19 – Londrina, PR, Brazil, 2023.

ID	Main gastrointestinal symptoms	Tests carried out	Association of gastrointestinal symptoms with COVID-19
Xu et al.^(35)^	Diarrhea (30%).	Tomography, blood count and RT-PCR.	Positive real-time RT-PCR results were observed in rectal swabs of children, which remain detectable after nasopharyngeal swabs become negative, suggesting that the GI tract may release virus and fecal-oral transmission may be possible.
Miller et al.^(36)^	Nausea (29.5%), vomiting (29.5%), and abdominal pain (29.5%).	PCR, erythrocyte sedimentation rate, albumin, ALT, AST, Ultrasound, resonance.	SARS-CoV-2 triggers the process of Multisystem Inflammatory Syndrome in Children and subsequent symptoms arising from this inflammation affect the GI tract.
Giacomet et al.^(37)^	Vomiting (22%), diarrhea (9.4%), and abdominal pain (6.3%).	Radiography.	The GI tract is a target for SARS-CoV-2 due to the expression of angiotensin-converting enzyme 2 being one of the main receptors for the virus.
Dooki et al.^(38)^	Anorexia (83.3%), nausea (38.9%), vomiting (38.9%), diarrhea (33.3), and abdominal pain (33.3).	ALT, AST, ALP, prothrombin, thromboplastin, TTP, INR, Albumin, Direct and Total Bilirubin, PCR, Sedimentation Rate, Erythrocytes, Tomography, Radiography, and Ultrasonography.	The interaction between ACE2 and the SARS-CoV-2 virus may occur due to the receptors being present in the gastrointestinal system, as well as to a cytokine storm and dysregulation of the intestinal flora through immunological mechanisms.
Souza et al.^(39)^	Fever (100%), abdominal pain (30%), and jaundice (15%).	Ultrasound, PCR-RT and blood count.	The emergence of gastrointestinal symptoms is due to the Coronavirus entering cells that use the ACE2 enzyme receptor. The receptor is found in large quantities in lung cells, but also in epithelial cells of the esophagus, ileum and colon.
Lo Vecchio et al.^(40)^	Diarrhea (87.7%), vomiting (60%), nausea (28.6%), anorexia (28.6%), pain and abdominal distension (20.3%).	Ferritin, ALT, Abdominal ultrasound, intra-abdominal fluid/tissue samples.	SARS-CoV-2 has direct action on cells, due to the abundant expression of ACE2 and TMPRSS2 binding receptors on the surface of enterocytes, facilitating the entry of RNA into the cell. The GI tract is a potential target of immune-mediated inflammatory response by SARS-CoV-2.
Berni Canani et al.^(41)^	Abdominal pain (90%), vomiting (90%), constipation (90%) and diarrhea (50%).	Cytology of epithelial samples, intestinal epithelial biopsy, PCR, IgG, IgM, AST, ALT, CBC.	In the small intestine, ACE2 expression is higher in children. The ACE2 receptor acts as a gateway for the virus and TMPRSS2 is used by SARS-CoV-2 to initiate the protein *spike,* and NRP1, a SARS-CoV-2 coreceptor, increases the virus’s ability to enter and enhances infection in host cells.
Farello et al.^(42)^	Abdominal pain (13.6%), rumination (2.7%), constipation (13.6%).	Rome III Criteria (For non-organic diseases).	The COVID-19 pandemic generated anxiety in healthy people and exacerbated pre-existing mental illnesses. Social restrictions represented a threat to the mental health of children and adolescents, being a source of stress that, combined with changes in routine, generates a trigger for the appearance of episodes of abdominal pathology and the recurrence of symptoms related to irritable bowel syndrome.
Stepan et al.^(43)^	Abdominal pain (60.9%).	Rome IV Criteria (For non-organic diseases).	Functional gastrointestinal disorders are mediated by stress; psychosocial health in the pandemic has had long-term repercussions. SARS-CoV-2 infection is justified by the gut-brain axis where they are connected, providing viral infection.
Vasichkina et al.^(44)^	Suppressed appetite (100%), abdominal pain (100%), semi-liquid stools (100%).	PCR, procalcitonin, ferritin, D-dimer, x-ray, echocardiogram.	Multisystem inflammatory syndrome in children can be caused after COVID-19 and after SARS-2-CoV-2 infection and long-COVID-19, with the persistence of the virus. The presence of comorbidities is a risk factor for the development of the syndrome.

* RT-PCR: Reverse Transcriptase Reaction-Polymerase Chain Reaction; GI tract: Gastrointestinal Tract; ALT: Alanine Aminotransferase; AST: aspartate aminotransferase; SARS-CoV-2: Severe acute respiratory syndrome coronavirus 2; ALP: Alkaline phosphatase; TTP: Thrombotic thrombocytopenic purpura; INR: International Standard Ratio; RNA: Ribonucleic Acid; IgM: Immunoglobulin M; IgG: Immunoglobulin G.

**Figure 2:**
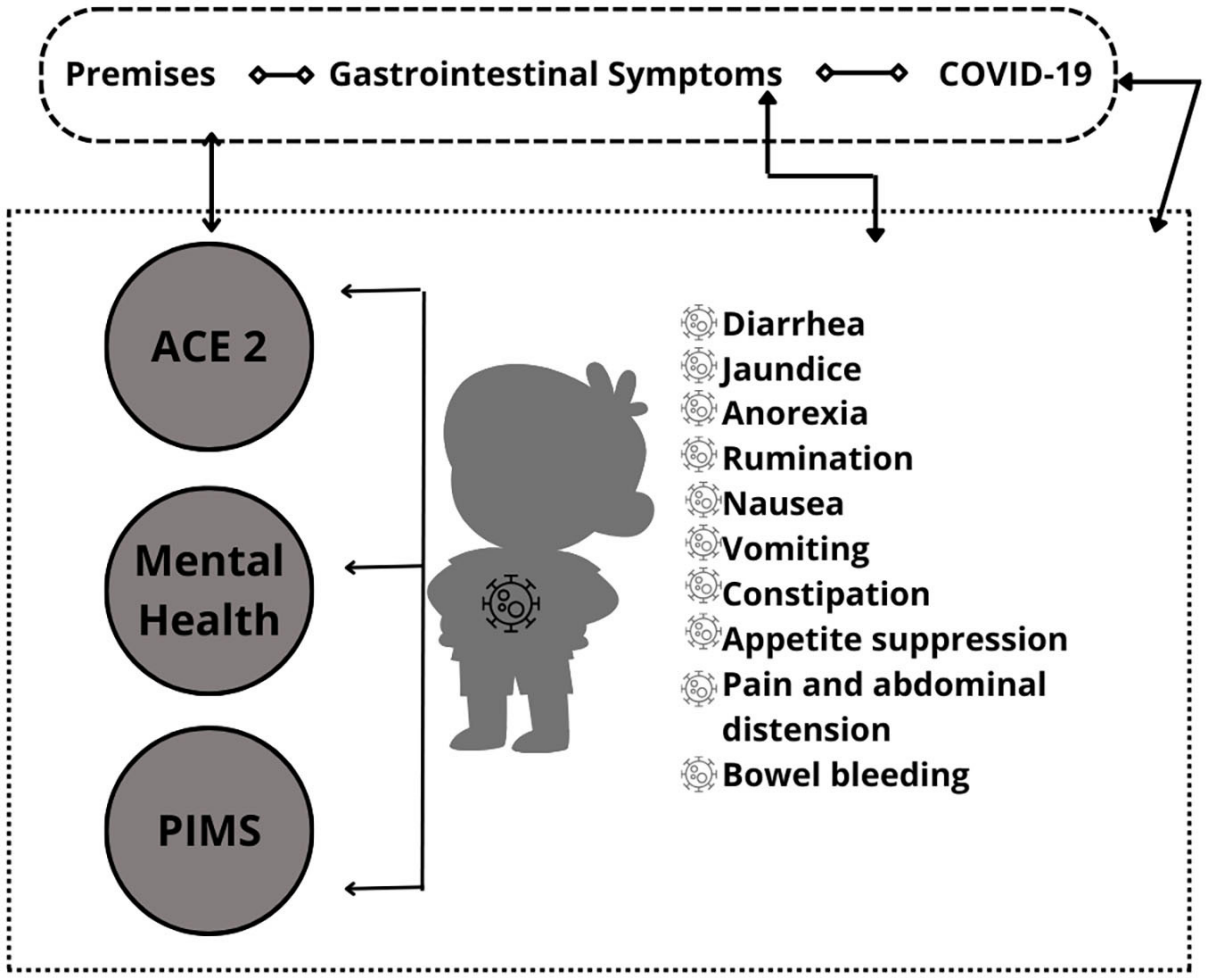
Gastrointestinal premises and symptoms presented by the pediatric population affected by COVID-19. Londrina, PR, Brazil, 2023.

The findings pointed to three premises, which present possible explanations of the clinical conditions and the clarification of the symptoms presented by the pediatric population, namely: the first premise points to the ACE 2 receptor (Angiotensin Converting Enzyme 2) which is found in the epithelial cells of the gastrointestinal tract and SARS-CoV-2 enters the cells via this route, giving rise to gastric manifestations. The second premise refers to gastrointestinal symptoms that are mediated by stress and the mental health of the pediatric population in the pandemic has suffered long-term repercussions. Thus, infection by the virus can be justified by the brain and intestine axis where the viral infection is triggered, generating destabilization of structures. The third premise is that COVID-19 develops the process of Pediatric Multisystem Inflammatory Syndrome (MIS-P), affecting the gastrointestinal tract and triggering symptoms such as diarrhea, vomiting, nausea, and abdominal pain (Figure 2).

## DISCUSSION

This scoping review covers gastrointestinal manifestations in the pediatric population resulting from COVID-19 infection and the mechanism by which the virus is capable of causing the disorder. COVID-19 is a systemic disease, which has been presenting gastrointestinal symptoms^(22,37)^ in the pediatric population as initial symptoms, which differentiates it from adults who, in general, have a high prevalence of respiratory symptoms^(39)^. The average number of pediatric patients who developed only gastrointestinal symptoms and no respiratory symptoms is 33.5%^(22,37)^. In this regard, the symptoms presented by the pediatric population diagnosed with COVID-19 are: diarrhea, anorexia, vomiting, nausea, abdominal pain and gastrointestinal bleeding^(36,45,46)^.

Of these symptoms, diarrhea was identified as the main sign in the absence of respiratory findings, in 22% of cases^(45)^ and its onset is described from 01 to 08 days after the onset of the disease, and with an average of 03 to 06 days, with a watery, yellowish characteristic and with a frequency of three to nine bowel movements per day^(47,48)^. Therefore, fecal-oral transmission should be considered, even if there are no gastrointestinal symptoms, thus preventing the spread of the virus through this route^(35,49,50)^.

The findings of the selected studies pointed to three premises about the relationships between COVID-19 and gastrointestinal symptoms in the pediatric population. The first assumption to be addressed is that there is intestinal impairment caused by the COVID-19 virus, as the ACE2 receptor is the cellular link for SARS-CoV-2^(51)^. An example of this is that the glycoprotein *spike* belonging to SARS-CoV-2 binds to the extracellular part of ACE2 in host cells, providing high levels of expression of the ACE2 receptor^(40,52)^.

The ACE2 receptor enzyme has an important function in regulating intestinal homeostasis. After this influence of the virus on cells occurs, the receptor’s activities are interrupted in the gastrointestinal tract, generating evident differences in its expression and causing gastrointestinal manifestations^(38,39)^.

Although the expression of SARS-CoV-2 mediators acts in different locations, such as in the intestinal epithelium and nasal epithelium, ACE1 and ACE2 are more expressed at the intestinal level in children than in adults, which explains why the pediatric population presents more evident gastrointestinal symptoms compared to adults^(41)^. In addition, transmembrane serine protease 2 (TMPRSS2), which also initiates the protein *spike* and acts as a co-receptor for SARS-CoV-2, is predominantly found in adults; neuropilin 1 (NRP1) has a high capacity to enhance infection in cells at the nasal level, another explanation for respiratory symptoms being more pronounced in the adult population^(41)^.

The second premise found in the results of this review is that functional gastrointestinal disorders may arise from symptomatic disorders without organic abnormalities, with psychosomatic origins^(53)^, that is, stress-sensitive disorders, which are gastrointestinal pathologies with multifactorial pathophysiology, the main cause being dysregulation of the gut-brain axis^(42)^.

It is believed that the pandemic led to changes in children’s routines, and several safety measures were taken to combat the virus, one of which was social distancing, which contributed to the manifestation of mental illnesses^(54)^. The main gastrointestinal findings related to functional gastrointestinal disorders are cyclic vomiting syndrome, functional constipation^(55)^, aerophagia, and abdominal pain^(43)^.

In this sense, following the premises set out in the studies, there is the third condition, the association of gastrointestinal symptoms caused by SARS-CoV-2 and Pediatric Multisystem Inflammatory Syndrome, highlighted by fever and bleeding, being evidenced in more severe presentations of the disease^(27,36,56)^. It can be observed that patients who were detected with the COVID-19 virus had gastrointestinal symptoms along with rashes, elevated inflammatory markers, and myocardial involvement^(57)^. Imaging exams are also complementary to intestinal inflammation resulting from MIS-P^(36)^.

Another issue imposed is that Long COVID-19, which is the second long-term infection caused by SARS-Cov-2, can develop polyorganic disorders^(58,59)^. It can be observed that reinfection is capable of generating some respiratory damage (21.2%), nervous disorders (16%), integumentary tissue (15%), gastrointestinal tract (13%), cardiovascular system (11%), as well as possible psychiatric symptoms (10%) and pathologies of the intestinal system (9%)^(60)^. Long COVID-19 is directly associated with the causes of MIS-P. Due to the persistence of the virus^(57)^, some symptoms can be expected in up to three months or more. Therefore, observation of patients must be carried out at future levels, even when detection of the virus by occupational diagnostic methods is negative^(60)^.

This way, special attention is required for patients who have gastrointestinal symptoms and a history of exposure to or infection with SARS-CoV-2, as data suggest that contact with the virus triggers MIS-P, and gastrointestinal symptoms are signs of this condition^(44)^.

It should be highlighted that the results presented contribute to formulating strategies for caring for this population, giving emphasis and importance to gastrointestinal symptoms when reported. Some limitations were found, such as the inclusion of only studies available in full and free of charge, which may have led to the exclusion of studies relevant to the proposed synthesis. Additionally, nine databases were used, which also limits the number of sources reviewed, and may result in the exclusion of potential studies that could contribute to elucidating the findings. There were also no procedures used to evaluate the evidence found.

## CONCLUSION

The gastrointestinal symptoms presented by the pediatric population are diarrhea, anorexia, vomiting, nausea, abdominal pain, and gastrointestinal bleeding, with diarrhea being the most prevalent symptom in this age group. The identification of gastrointestinal symptoms in the pediatric population affected by COVID-19 can assist in the clinical approach and in the discussion of care and treatment management for these children.

The evidence synthesis provided three assumptions that guide the origin of the symptoms. One of them is the ACE2 receptor, which is found in the epithelial cells of the gastrointestinal tract and favors the entry of SARS-CoV-2 into the cells, causing infection and generating gastric manifestations. Also, the mental health of the pediatric population was affected by the pandemic and the restrictions imposed, causing disorders of the gut-brain axis, thus affecting gastrointestinal manifestations. Furthermore, the virus is capable of triggering gastrointestinal symptoms due to its ability to develop the process of multisystem inflammatory syndrome.
